# Octenidine-Based Versus Chlorhexidine-Based Preoperative Skin Antisepsis in Primary Hip and Knee Arthroplasty

**DOI:** 10.1016/j.artd.2026.102113

**Published:** 2026-08-12

**Authors:** Abialevich Artsiom, Benkovich Vadim

**Affiliations:** a Department of Orthopedic, Assuta Medical Center, Tel Aviv-Yafo, Israel; b Department of Orthopedic, Soroka University Medical Center, Be’er Sheva, Israel; c Surgical Division, Faculty of Health Sciences, Ben-Gurion University of the Negev, Be’er Sheva, Israel

**Keywords:** Octenidine, Chlorhexidine, Total hip arthroplasty, Total knee arthroplasty, Surgical site infection

## Abstract

**Background:**

Surgical site infection (SSI) and periprosthetic joint infection (PJI) remain major complications after primary total hip arthroplasty (THA) and total knee arthroplasty (TKA). Preoperative antisepsis bundles are essential components of infection-prevention pathways; however, comparative data on octenidine-based vs chlorhexidine-based protocols remain limited. This study compared infectious and wound-related outcomes between these bundles in primary THA and TKA.

**Methods:**

This retrospective cohort study included 1767 consecutive patients who underwent primary THA or TKA between March 2015 and September 2024. Patients were stratified by antisepsis bundle: chlorhexidine-based group, 867 patients; and octenidine-based group, 900 patients. Baseline characteristics, American Society of Anesthesiologists class, comorbidities, procedure type, wound complications, SSI, PJI, readmission, and revision surgery were analyzed. Primary outcomes were 30-day superficial SSI, 90-day deep SSI/PJI, and 1-year PJI. Secondary outcomes included composite infectious and wound-related events. Between-group comparisons and adjusted analyses were performed where permitted.

**Results:**

Baseline characteristics were comparable. Thirty-day superficial SSI occurred in 0.8% of chlorhexidine patients and 1.1% of octenidine patients (Relative risk [RR], 1.38; 95% confidence interval (CI), 0.53-3.61; *P* = .682). Ninety-day deep SSI/PJI occurred in 0.1% vs 0.0% (RR, 0.32; 95% CI, 0.01-7.80; *P* = .491), and 1-year PJI in 0.1% vs 0.0% (RR, 0.32; 95% CI, 0.01-7.80; *P* = .491). Composite infectious events were similar (0.9% vs 1.1%; RR, 1.20; 95% CI, 0.47-3.04; *P* = .875). Composite wound-related events occurred in 45.1% vs 41.7% (RR, 0.92; 95% CI, 0.83-1.03; *P* = .159).

**Conclusions:**

Octenidine-based preoperative antisepsis showed outcomes comparable to chlorhexidine-based antisepsis in primary THA and TKA.

## Introduction

Total hip arthroplasty (THA) and total knee arthroplasty (TKA) are increasingly performed worldwide and are projected to continue rising in volume over the coming decades [1]. Although these procedures are highly successful, surgical site infection (SSI) and periprosthetic joint infection (PJI) remain among the most serious complications after primary total joint arthroplasty [2]. Even with relatively low reported infection rates, SSI and PJI are associated with major clinical consequences, including prolonged hospitalization, revision surgery, increased healthcare costs, impaired recovery, and increased mortality [3,4].

Prevention of infection after THA and TKA requires a multimodal perioperative approach, including patient optimization, antibiotic prophylaxis, meticulous surgical technique, and effective reduction of bacterial burden before surgery [[5], [6], [7]]. Preoperative skin antisepsis bundles are therefore an important component of infection-prevention pathways. Chlorhexidine-based protocols are widely used, whereas octenidine-based protocols have been introduced as an alternative in some clinical settings. However, comparative outcome data between these 2 antisepsis strategies in primary THA and TKA remain limited. Chlorhexidine-based products are widely used in perioperative antisepsis protocols, whereas octenidine-based products are used in some European and institution-specific infection-prevention pathways as an alternative broad-spectrum antiseptic option. Therefore, comparative clinical data between these bundles are relevant for institutions considering alternative preoperative decolonization protocols.

The purpose of this study was to compare infectious and wound-related outcomes after octenidine-based vs chlorhexidine-based preoperative skin antisepsis bundles in primary THA and TKA. We hypothesized that octenidine-based antisepsis would demonstrate outcomes comparable to those of chlorhexidine-based protocols.

## Material and methods

This retrospective cohort study included 1767 patients who underwent primary THA or TKA between March 1, 2015, and September 30, 2024. Patients were stratified according to the preoperative antisepsis bundle used before surgery: a chlorhexidine-based bundle group comprising 867 patients and an octenidine-based bundle group comprising 900 patients. The study aimed to compare postoperative infectious and wound-related outcomes between the 2 institutional preoperative skin antisepsis protocols. No patients were excluded from this final analyzed cohort.

Eligible patients were adults undergoing primary THA or TKA for degenerative hip or knee disease [8,9]. Patients with active infection, previous infection of the operative joint, active wounds of the operative limb, and inflammatory arthropathy were not included in the study database and therefore were not counted as exclusions from the main cohort. Preoperative optimization was performed according to institutional practice, including assessment of modifiable risk factors such as diabetes control, obesity, anemia, hypoalbuminemia, and smoking status.

Collected variables included age, sex, body mass index, American Society of Anesthesiologists (ASA) physical status classification, diabetes mellitus, smoking status, type of arthroplasty, antisepsis bundle, length of hospital stays, wound complications, superficial SSI, deep infection or periprosthetic joint infection, readmission, revision surgery, and mortality when available. ASA class was analyzed as 1 categorical variable with 3 levels, and 1 overall *P* value was calculated for comparison between groups.

The preoperative antisepsis protocol was implemented as a bundle rather than as a single isolated antiseptic agent. In both cohorts, patients began the protocol in a home-based manner 5 days before hospitalization and surgery, according to written institutional instructions. The chlorhexidine-based bundle consisted of whole-body washing with Septal Scrub (Teva, Israel), a chlorhexidine gluconate 4% weight per volume external solution, together with abroad-spectrum antiseptic containing povidone-iodine (PVP-I) - Iso-Betadine 5% for nasal decolonization according to the institutional protocol used during that period. The octenidine-based bundle consisted of whole-body and hair washing with Octenisan wash lotion (Schülke & Mayr GmbH, Norderstedt, Germany), an Octenidine hydrochloride-containing wash lotion, together with the octenidine-based nasal gel, used for patient decolonization in our institutional pathway. Patients were instructed to apply the body-washing product daily during the 5-day preoperative period, using it undiluted on wet skin or with a damp washcloth, with rinsing after the recommended contact time, and to complete the nasal/mucosal component according to the same institutional decolonization schedule. Standard perioperative antibiotic prophylaxis, operating room skin preparation, thromboprophylaxis, postoperative dressing care, and follow-up pathways were otherwise applied similarly in both groups. THA and TKA were performed using standard arthroplasty techniques. TKA was generally performed through a midline incision and medial parapatellar approach, while THA was performed using the institutional standard approach according to surgeon preference. Anesthesia type and use of regional blocks were determined by the anesthesiology team.

The primary outcome was postoperative SSI. Infectious outcomes included 30-day superficial SSI, 90-day deep SSI or periprosthetic joint infection, and 1-year periprosthetic joint infection when available. Secondary outcomes included wound drainage, dehiscence, hematoma or seroma, other wound-related complications, readmission, revision surgery, and length of stay.

Postoperative field hematoma was included as a wound-related outcome because it may influence early wound drainage, local wound healing, clinical surveillance for infection, and the interpretation of postoperative wound events. Its inclusion was not based on an assumed direct biological effect of either antiseptic wash on hematoma formation, but rather on its relevance as a postoperative wound-field variable within the overall wound-complication profile.

Continuous variables were reported as means with standard deviations or medians with interquartile ranges, as appropriate. Categorical variables were reported as frequencies and percentages. Between-group comparisons were performed using *t* tests or Welch’s *t* tests for continuous variables and chi-square or Fisher’s exact tests for categorical variables. Risk ratios with 95% confidence intervals (CIs) were calculated for binary outcomes when appropriate. Multivariable logistic regression was used for clinically relevant endpoints when event numbers permitted. As a sensitivity analysis, we performed the wound-event analysis using a modified composite endpoint excluding hematoma, defined as persistent wound drainage during the first postoperative week or superficial SSI within 30 days. A two-sided *P* value below 0.050 was considered statistically significant.

## Results

**Figure 1:**
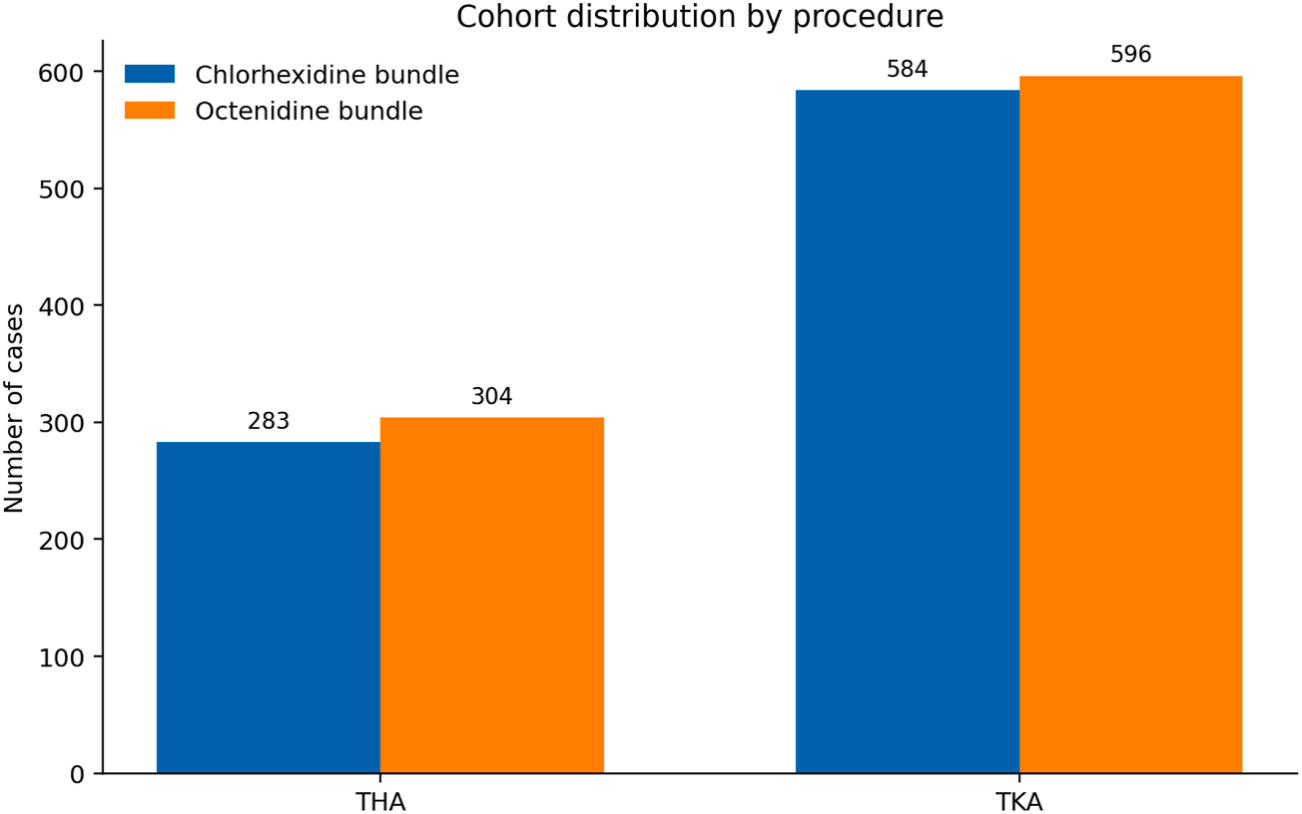
Cohort distribution by procedure and antisepsis bundle.

The final cohort included 1767 primary arthroplasty cases: 867 in the chlorhexidine-based cohort and 900 in the octenidine-based cohort. Baseline and perioperative characteristics are summarized in Table 1, and procedure distribution is shown in Figure 1. Overall, 1180 cases (66.8%) were TKA and 587 (33.2%) were THA. Baseline characteristics were well balanced between groups. ASA class was analyzed as a single three-level categorical variable (overall *P* = .843; Table 1); exploratory binary tests were ASA 1 *P* = .978, ASA 2 *P* = .634, and ASA 3 *P* = .600.

**Table 1:** Baseline and perioperative characteristics.

Characteristic	Overall (N = 1767)	Chlorhexidine bundle (n = 867)	Octenidine bundle (n = 900)	*P* value
TKA procedure	1180 (66.8%)	584 (67.4%)	596 (66.2%)	.648
Cementless fixation	1184 (67.0%)	576 (66.4%)	608 (67.6%)	.653
Age, years	69.8 ± 9.8	69.7 ± 9.8	69.9 ± 9.8	.652
Female sex	1027 (58.1%)	488 (56.3%)	539 (59.9%)	.137
BMI, kg/m^2^	31.5 ± 3.1	31.5 ± 3.2	31.5 ± 3.0	.933
ASA class				.843
ASA 1	619 (35.0%)	304 (35.1%)	315 (35.0%)	
ASA 2	618 (35.0%)	308 (35.5%)	310 (34.4%)	
ASA 3	530 (30.0%)	255 (29.4%)	275 (30.6%)	
Charlson Comorbidity Index	4.3 ± 2.2	4.3 ± 2.2	4.2 ± 2.2	.117
Chronic anticoagulation therapy	572 (32.4%)	287 (33.1%)	285 (31.7%)	.552
Diabetes mellitus	424 (24.0%)	201 (23.2%)	223 (24.8%)	.466
HbA1c, %	5.5 ± 0.9	5.5 ± 0.9	5.5 ± 0.9	.190
Current smoking	124 (7.0%)	59 (6.8%)	65 (7.2%)	.803
Immunosuppression	9 (0.5%)	4 (0.5%)	5 (0.6%)	1.000
Positive MRSA screening	18 (1.0%)	6 (0.7%)	12 (1.3%)	.269
Cefazolin prophylaxis	1749 (99.0%)	861 (99.3%)	888 (98.7%)	.269
Complete overall bundle compliance	1732 (98.0%)	845 (97.5%)	887 (98.6%)	.139
Procedure duration, minutes	88.9 ± 5.8	88.8 ± 5.8	89.0 ± 5.8	.606
Length of stay, hours	15.3 ± 5.1	15.5 ± 5.2	15.1 ± 5.0	.126
Discharge home	1742 (98.6%)	857 (98.8%)	885 (98.3%)	.477

Values are mean ± standard deviation or n (%).

ASA, American Society of Anesthesiologists; BMI, body mass index; CCI, Charlson Comorbidity Index; THA, total hip arthroplasty; TKA, total knee arthroplasty.

Procedure duration was similar between cohorts (88.8 ± 5.8 min vs 89.0 ± 5.8 min; *P* = .606). Length of stay was also comparable (15.5 ± 5.2 h vs 15.1 ± 5.0 h; *P* = .126), and most patients were discharged home in both cohorts.

**Figure 2:**
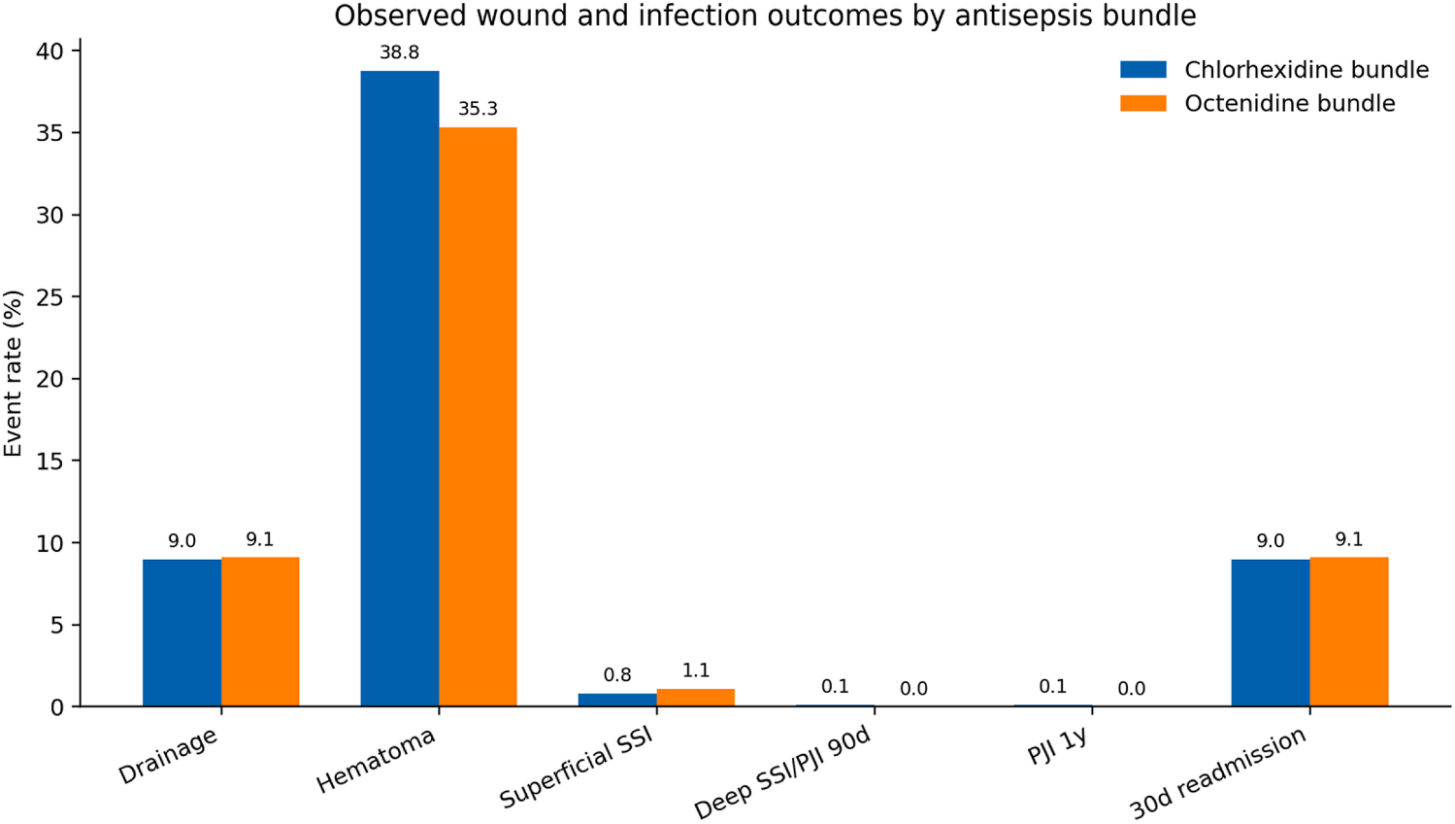
Observed wound and infection outcome rates by antisepsis bundle.

**Figure 3:**
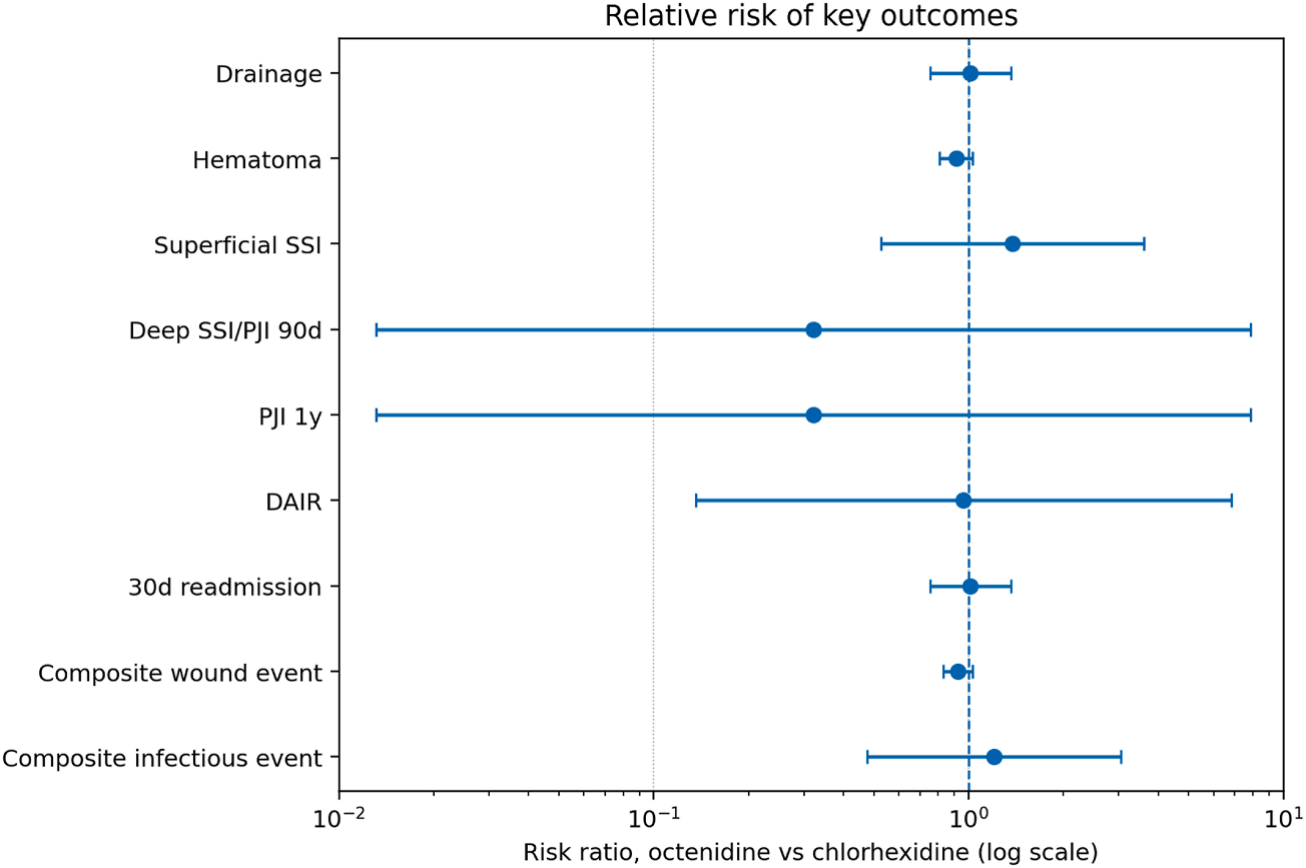
Forest plot of risk ratios for key outcomes. Risk ratios compare octenidine-based with chlorhexidine-based bundles.

Infectious outcomes are summarized in Table 2, with observed outcome rates shown in Figure 2 and unadjusted risk ratios shown in Figure 3. Superficial SSI within 30 days occurred in 7/867 cases (0.8%) in the chlorhexidine-based bundle cohort and 10/900 cases (1.1%) in the octenidine-based bundle cohort (risk ratio 1.38, 95% CI 0.53-3.60; *P* = .682). Deep SSI/PJI within 90 days and PJI within 1 year were rare. One deep SSI/PJI and one 1-year PJI were documented in the chlorhexidine cohort, compared with no events in the octenidine cohort (both *P* = .491). Culture-positive infection and infection-related revision were each documented in 1 case in the chlorhexidine group and in no cases in the octenidine group (Table 2; Fig. 3).

**Table 2:** Wound, SSI, PJI, readmission, and revision outcomes.

Outcome	Overall (N = 1767)	Chlorhexidine bundle (n = 867)	Octenidine bundle (n = 900)	Risk ratio (95% CI)^a^	*P* value
Persistent wound drainage during first postoperative week	160 (9.1%)	78 (9.0%)	82 (9.1%)	1.01 (0.75-1.36)	.999
Postoperative field hematoma	654 (37.0%)	336 (38.8%)	318 (35.3%)	0.91 (0.81-1.03)	.150
Superficial SSI within 30 days	17 (1.0%)	7 (0.8%)	10 (1.1%)	1.38 (0.53-3.60)	.682
Antibiotic treatment for superficial SSI	17 (1.0%)	7 (0.8%)	10 (1.1%)	1.38 (0.53-3.60)	.682
Deep SSI or PJI within 90 days	1 (0.1%)	1 (0.1%)	0 (0.0%)	0.32 (0.01-7.87)	.491
PJI within 1 year	1 (0.1%)	1 (0.1%)	0 (0.0%)	0.32 (0.01-7.87)	.491
Culture-positive infection	1 (0.1%)	1 (0.1%)	0 (0.0%)	0.32 (0.01-7.87)	.491
Reoperation/revision for infection within 1 year	1 (0.1%)	1 (0.1%)	0 (0.0%)	0.32 (0.01-7.87)	.491
DAIR performed	4 (0.2%)	2 (0.2%)	2 (0.2%)	0.96 (0.14-6.82)	1.000
Readmission to ER/ambulatory clinic within 30 days	160 (9.1%)	78 (9.0%)	82 (9.1%)	1.01 (0.75-1.36)	.999
Readmission to ER/hospitalization within 90 days	4 (0.2%)	2 (0.2%)	2 (0.2%)	0.96 (0.14-6.82)	1.000
Composite wound event^b^	766 (43.4%)	391 (45.1%)	375 (41.7%)	0.92 (0.83-1.03)	.159
Composite infectious event^c^	18 (1.0%)	8 (0.9%)	10 (1.1%)	1.20 (0.48-3.04)	.875
Time to superficial SSI diagnosis, days^d^	8.0 (5.0-11.0)	11.0 (6.5-11.0)	7.5 (5.0-8.8)	—	.351

DAIR, debridement, antibiotics, and implant retention; ER, emergency room.

a Risk ratio compares octenidine-based bundle with chlorhexidine-based bundle. Continuity correction was used for outcomes with 0 cells.

b Composite wound event: drainage, hematoma, or superficial SSI.

c Composite infectious event: superficial SSI, deep SSI/PJI, 1-year PJI, or infection-related revision.

d Among cases with superficial SSI.

Wound-related outcomes are presented in Table 2, Figures 2 and 3. Persistent wound drainage during the first postoperative week was similar between groups (78/867 [9.0%] vs 82/900 [9.1%]; *P* = .999). Hematoma was documented in 336 cases (38.8%) in the chlorhexidine cohort and 318 cases (35.3%) in the octenidine cohort (*P* = .150). The composite wound event rate was 391/867 (45.1%) vs 375/900 (41.7%) (*P* = .159).

**Figure 4:**
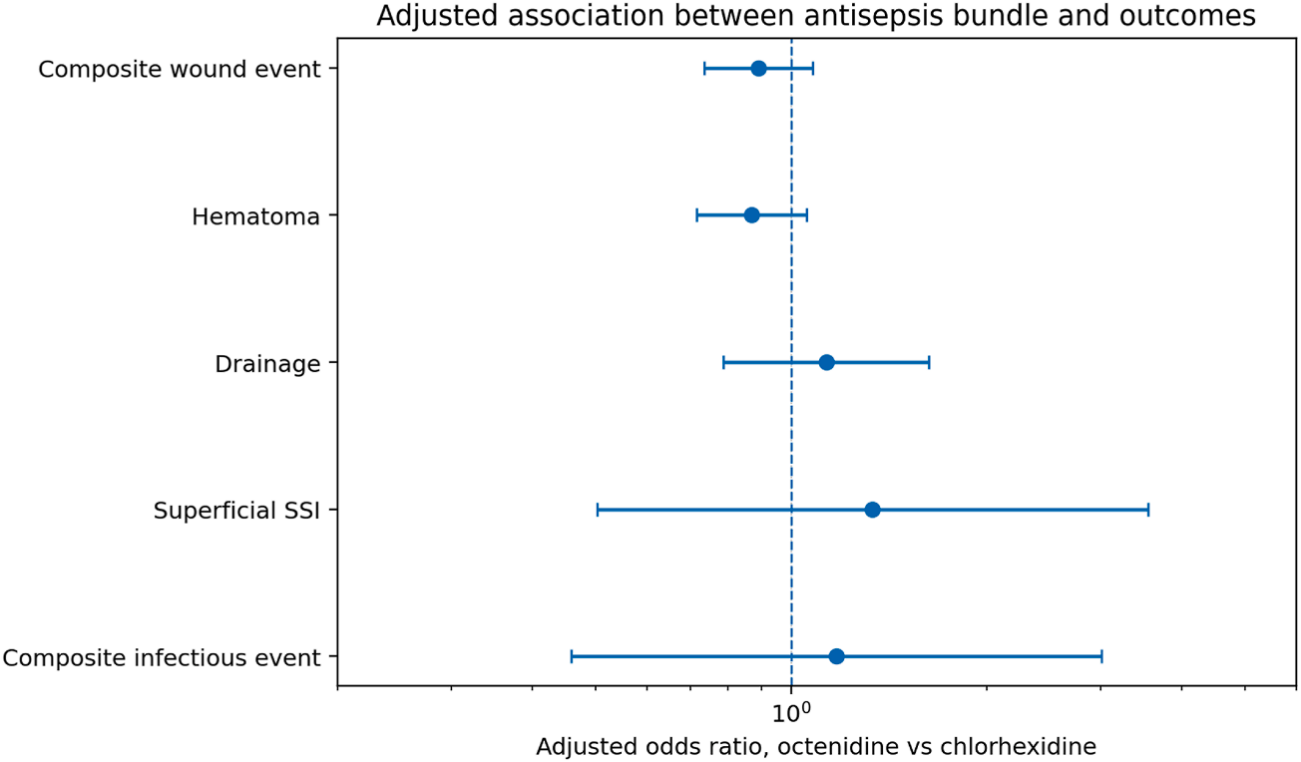
Adjusted odds ratios for selected outcomes. Odds ratios compare octenidine-based with chlorhexidine-based bundles.

Adjusted logistic regression models are presented in Table 3, and adjusted odds ratios (ORs) for selected endpoints are displayed in Figure 4. After adjustment for age, sex, body mass index, procedure type, diabetes, ASA class, and Charlson Comorbidity Index, the octenidine-based bundle was not independently associated with composite wound events (adjusted OR 0.89, 95% CI 0.73-1.08; *P* = .239), hematoma (adjusted OR 0.87, 95% CI 0.72-1.06; *P* = .159), drainage (adjusted OR 1.13, 95% CI 0.79-1.63; *P* = .505), superficial SSI (adjusted OR 1.34, 95% CI 0.50-3.54; *P* = .562), or composite infectious events (adjusted OR 1.17, 95% CI 0.46-3.01; *P* = .739). In the sensitivity analysis excluding hematoma from the composite wound endpoint, events occurred in 85/867 patients (9.8%) in the chlorhexidine cohort and 91/900 patients (10.1%) in the octenidine cohort. After adjustment, octenidine was not associated with increased risk of this modified endpoint (adjusted OR 1.15, 95% CI 0.82-1.62; *P* = .426).

**Table 3:** Adjusted logistic regression models.

Outcome	Adjusted OR for octenidine vs chlorhexidine (95% CI)	*P* value	Model notes
Composite wound event	0.89 (0.73-1.08)	.239	Adjusted for age, sex, BMI, operation type, diabetes, ASA class, and CCI.
Composite wound event excluding hematoma	1.15 (0.82-1.62)	.426	Sensitivity analysis; drainage or superficial SSI only; adjusted as above.
Postoperative field hematoma	0.87 (0.72-1.06)	.159	Adjusted for age, sex, BMI, operation type, diabetes, ASA class, and CCI.
Persistent wound drainage	1.13 (0.79-1.63)	.505	Adjusted for age, sex, BMI, operation type, diabetes, ASA class, and CCI.
Superficial SSI within 30 days	1.34 (0.50-3.54)	.562	Exploratory model; low event count.
Composite infectious event	1.17 (0.46-3.01)	.739	Exploratory model; low event count.

Adjusted ORs compare the octenidine-based bundle with the chlorhexidine-based bundle. Models adjusted for age, sex, BMI, procedure type, diabetes, ASA class, and CCI. The modified composite wound event excluding hematoma included persistent wound drainage during the first postoperative week or superficial SSI within 30 days.

Procedure-specific exploratory outcomes are shown in Table 4. In THA cases, the octenidine-based bundle was associated with lower unadjusted rates of composite wound events (134 (47.3%) vs 116 (38.2%); *P* = .030) and hematoma (113 (39.9%) vs 93 (30.6%); *P* = .022), whereas superficial SSI and deep SSI/PJI did not differ significantly. In TKA cases, no significant differences were observed for composite wound events, drainage, hematoma, superficial SSI, or deep SSI/PJI (Table 4).

**Table 4:** Procedure-specific exploratory outcomes.

Procedure subgroup and outcome	Chlorhexidine bundle	Octenidine bundle	*P* value
THA			
Composite wound event	134 (47.3%)	116 (38.2%)	.030
Postoperative field hematoma	113 (39.9%)	93 (30.6%)	.022
Persistent wound drainage	26 (9.2%)	27 (8.9%)	1.000
Superficial SSI within 30 days	5 (1.8%)	2 (0.7%)	.271
Deep SSI/PJI within 90 days	1 (0.4%)	0 (0.0%)	.482
TKA			
Composite wound event	257 (44.0%)	259 (43.5%)	.895
Postoperative field hematoma	223 (38.2%)	225 (37.8%)	.926
Persistent wound drainage	52 (8.9%)	55 (9.2%)	.926
Superficial SSI within 30 days	2 (0.3%)	8 (1.3%)	.108
Deep SSI/PJI within 90 days	0 (0.0%)	0 (0.0%)	1.000

## Discussion

In this retrospective cohort study of primary THA and TKA, octenidine-based preoperative antisepsis was associated with postoperative infectious outcomes comparable to those observed after chlorhexidine-based antisepsis. The rates of 30-day superficial SSI, 90-day deep SSI or PJI, 1-year PJI, and the composite infectious endpoint did not differ significantly between groups. Similarly, the overall rate of composite wound events was not significantly different between the chlorhexidine-based and octenidine-based cohorts. The hematoma findings should be interpreted cautiously. Postoperative field hematoma was analyzed as part of the broader wound-related outcome profile because it may influence early drainage, local wound healing, postoperative surveillance, and concern for subsequent infectious complications. However, hematoma formation was not assumed to be directly or biologically related to the preoperative antiseptic bundle. Accordingly, the lower hematoma rate observed in the exploratory THA subgroup should be considered hypothesis-generating and may reflect procedure-related factors, temporal changes in perioperative practice, anticoagulation management, surgical technique, or documentation patterns rather than an antiseptic-specific effect.

These findings suggest that, within a standardized arthroplasty infection-prevention pathway, an octenidine-based preoperative bundle may represent a clinically reasonable alternative to a chlorhexidine-based protocol.

SSI and PJI remain serious complications after TKA and THA despite modern perioperative protocols. Previous studies have reported SSI rates after TKA ranging from approximately 0.5% to 2%, with Parvizi et al. [10] reporting an infection rate of 0.8% in a large TKA cohort. Infection rates after THA are similarly variable, with Bozic et al. [11] reporting a PJI incidence of 1.09% after primary THA and identifying several patient-related risk factors. In the present study, the observed infection rates were low in both cohorts and consistent with contemporary arthroplasty literature. Importantly, the absence of a significant difference between the 2 antisepsis protocols suggests that the transition to an octenidine-based protocol did not increase early infectious risk.

Current SSI-prevention strategies emphasize a multimodal approach rather than reliance on a single intervention. Guidelines and expert recommendations from surgical and infection–prevention organizations support preoperative patient cleansing, appropriate antimicrobial prophylaxis, perioperative normothermia, glycemic control, careful skin preparation, and standardized operative protocols [[12], [13], [14]]. Chlorhexidine-based products are widely used in this context, and chlorhexidine cloths have been associated with reduced bacterial burden and lower SSI risk in arthroplasty settings [15]. Alcohol-based antiseptic preparations also provide effective reduction of resident skin flora, although fire risk has been described when alcohol-containing preparations are used improperly with electrocautery [16,17]. Iodophor-based preparations remain another commonly used option, but comparative orthopaedic literature has suggested that alcoholic chlorhexidine may be superior to povidone-iodine for reducing bacterial colonization in clean orthopedic surgery [18,19].

Within a standardized arthroplasty infection-prevention pathway, several measures may contribute to reducing postoperative infectious risk, including assessment of potential preoperative infection sources such as dental disease, appropriate timing and duration of antibiotic prophylaxis, and selected intraoperative adjuncts such as antimicrobial incision drapes and triclosan-coated sutures [[20], [21], [22], [23]]. The rationale for using octenidine-based washes is mainly institutional and practical. Although chlorhexidine-based products remain widely used in perioperative antisepsis pathways, octenidine may be preferred in some centers because of local formulary availability, infection-control policy, compatibility with existing decolonization protocols, and its broad antimicrobial spectrum. Octenidine-based protocols may also be considered in settings where methicillin-resistant Staphylococcus aureus (MRSA) or multidrug-resistant organism decolonization is emphasized, or when chlorhexidine intolerance is a clinical concern. In this context, the present study provides clinically relevant comparative data for institutions considering octenidine-based bundles as an alternative to chlorhexidine-based protocols within a standardized arthroplasty infection-prevention pathway.

The present study adds to this literature by focusing on octenidine-based preoperative antisepsis in primary arthroplasty. Octenidine has broad antimicrobial activity against Gram-positive and Gram-negative organisms and has been reported to be effective against clinically relevant organisms, including MRSA-related colonization [24,25]. Its nonspecific antimicrobial mechanism may reduce the likelihood of resistance selection, which has been supported in experimental studies evaluating low-level MRSA exposure [26]. Brill et al. [27] reported residual antiseptic efficacy of octenidine comparable to chlorhexidine in alcoholic solutions, and Hachenberg et al. [28] described the feasibility and clinical utility of preoperative octenidine application in a surgical population. The current findings are consistent with these observations, showing no signal of increased SSI or PJI after implementation of an octenidine-based bundle.

Both chlorhexidine-based and octenidine-based antisepsis protocols may be associated with local cutaneous intolerance, including erythema, rash or contact dermatitis, pruritus, dryness or peeling, and, rarely, allergic reactions. These reactions may influence patient comfort and compliance with multiday preoperative home-based decolonization protocols. In the present study, antiseptic-related skin reactions were not systematically collected as a predefined outcome; therefore, no comparative conclusion can be drawn regarding tolerability or patient compliance between the 2 bundles. This represents an important area for future prospective studies, in which patient-reported symptoms, protocol adherence, and reasons for incomplete use should be assessed alongside infectious and wound-related outcomes.

A key strength of the current study is the relatively large real-world cohort including both primary THA and TKA cases over a long study period. In addition, the analysis evaluated several clinically relevant endpoints, including superficial SSI, deep SSI/PJI, 1-year PJI, composite infectious events, wound-related complications, readmission, and revision surgery. The comparison of baseline characteristics also allowed evaluation of cohort balance, including ASA class as a single categorical variable across all levels rather than as isolated binary comparisons. This approach better reflects the clinical meaning of ASA status and avoids overinterpretation of individual ASA subgroups.

Based on local institutional procurement prices, the estimated per-patient cost of the chlorhexidine-based bundle was 44.85 USD compared with 54.80 USD for the octenidine-based bundle, corresponding to a difference of 9.95 USD per patient. Because infectious and wound-related outcomes were comparable between groups in the present cohort, these data should be interpreted as a cost-differential or cost-minimization consideration rather than as a formal cost-effectiveness analysis.

Because hematoma may not be directly related to antisepsis choice, we performed a sensitivity analysis excluding hematoma from the wound composite. The findings remained unchanged, supporting that the wound-related results were not driven by hematoma events.

Several limitations should be acknowledged. First, the retrospective design introduces potential selection bias and limits causal inference. Second, the antisepsis protocol was determined by institutional practice rather than random allocation, and unmeasured temporal or practice-related changes may have influenced outcomes. Third, deep infectious events were rare, limiting the statistical power for adjusted modeling of PJI-specific endpoints. Fourth, although the study reflects routine clinical practice of consecutive cases, adherence to all components of the antisepsis bundle may not have been fully measurable in every patient. In addition, minor antiseptic-related cutaneous reactions—such as erythema, pruritus, rash/contact dermatitis, dryness, peeling, or patient-reported intolerance—were not systematically collected; therefore, the study cannot compare tolerability, allergic reactions, or compliance between the chlorhexidine-based and octenidine-based bundles. Finally, the study compared bundle-based protocols rather than isolated antiseptic agents; therefore, the findings should be interpreted as reflecting the clinical performance of complete preoperative antisepsis pathways rather than octenidine or chlorhexidine alone.

Despite these limitations, the findings support the safety and clinical comparability of octenidine-based preoperative antisepsis in primary THA and TKA. In a contemporary arthroplasty cohort, octenidine-based preparation was not associated with higher rates of SSI, PJI, or wound-related complications than chlorhexidine-based preparation. These results may be useful for institutions considering alternative antisepsis protocols within comprehensive arthroplasty infection-prevention programs.

## Conclusions

In this retrospective cohort study of patients undergoing primary THA and TKA, octenidine-based preoperative skin antisepsis demonstrated postoperative infectious and wound-related outcomes comparable to those of a chlorhexidine-based bundle. No significant differences were observed in 30-day superficial SSI, 90-day deep SSI or PJI, 1-year PJI, composite infectious events, or composite wound complications. These findings suggest that octenidine-based preoperative antisepsis may be a safe and effective alternative to chlorhexidine-based protocols within a standardized primary arthroplasty infection-prevention pathway. Further prospective studies are warranted to confirm these findings and to determine whether specific patient subgroups may benefit from 1 antisepsis strategy over another.

## CRediT authorship contribution statement

**Abialevich Artsiom:** Writing – original draft, Software, Methodology, Data curation. **Benkovich Vadim:** Writing – review & editing, Supervision, Project administration, Methodology.

## Conflicts of interest

The authors declare there are no conflicts of interest.

For full disclosure statements refer to https://doi.org/10.1016/j.artd.2026.102113.

## Appendix A Supplementary data

Conflict of Interest Statement for Vadim

Conflict of Interest Statement for Artsiom
