# Supplementary material for: Octenidine-Based Versus Chlorhexidine-Based Preoperative Skin Antisepsis in Primary Hip and Knee Arthroplasty
Source: Arthroplast Today. 2026 Aug 12;40:102113. doi: 10.1016/j.artd.2026.102113 (PMC13486999; doi:10.1016/j.artd.2026.102113)
Supplement: Supplementary file 2 [file mmc2.docx]

# CONFLICT OF INTEREST STATEMENT

***American Association of Hip and Knee Surgeons***

(Adopted from the American Academy of Orthopaedic Surgeons disclosure statement)

The following form **must be filled out completely and submitted by each author (example, 6 authors, 6 forms).**

**All items require a response. If there is no relevant disclosure for a given item, enter "*None*.”**

Manuscript Title: Octenidine-Based Versus Chlorhexidine-Based Preoperative Skin Antisepsis in Primary Hip and Knee Arthroplasty

1.	Royalties from a company or supplier (The following conflicts were disclosed) - none

2.	Speakers bureau/paid presentations for a company or supplier (The following conflicts were disclosed) - none

3A.	Paid employee for a company or supplier (The following conflicts were disclosed)	- none

3B.	Paid consultant for a company or supplier (The following conflicts were disclosed)	- none

3C.	Unpaid consultants for a company or supplier (The following conflicts were disclosed)	- none

4.	Stock or stock options in a company or supplier (The following conflicts were disclosed)	- none

5.	Research support from a company or supplier as a Principal Investigator (The following conflicts were disclosed)	- none

6.	Other financial or material support from a company or supplier (The following conflicts were disclosed)	- none

7.	Royalties, financial or material support from publishers (The following conflicts were disclosed)	- none

8.	Medical/Orthopaedic publications editorial/governing board (The following conflicts were disclosed) - none

9.	Board member/committee appointments for a society (The following conflicts were disclosed) - none

**Each author must sign AND print or type his/her name, date and submit a separate form**

In addition, one BLINDED Conflict of Interest form (no author names used) should be submitted per manuscript with all author disclosures.

**Abialevich Artsiom**	28.05.2026

Author Name (Print or Type)	Author Signature	Date
